# Systematic review of the effects of neuromuscular electrical stimulation in post-coronavirus disease

**DOI:** 10.4102/sajp.v81i1.2132

**Published:** 2025-08-30

**Authors:** Nathali Carvajal-Tello, Alejandro Segura-Ordóñez, Harry García-Muñoz, Lida J. Sánchez-Montoya, Luisa M. Cambindo-Larrahondo, Valentina Muñoz-Chaux, Johana P. Barahona-Guzmán, Andrés F. Caballero-Lozada

**Affiliations:** 1 Health and Movement Research Group, Faculty of Health Sciences, Universidad Santiago de Cali, Cali, Colombia; 2 Department of Anesthesiology and Resuscitation, Faculty of Health Sciences, Universidad del Valle, Cali, Colombia; 3 Department of Sport Science, Indervalle, Cali, Colombia; 4 Universidad San Martin, Cali, Colombia; 5 Intensive Care Unit, Hospital Universitario del Valle, Cali, Colombia; 6 Department of Anesthesiology, Hospital Universitario del Valle, Cali, Colombia; 7 Intensive Care Unit, Hospital San Jose de Buga, Buga, Colombia

**Keywords:** neuromuscular electrical stimulation, post-acute COVID-19 syndrome, muscle strength, therapeutic electric stimulation, functional independence, COVID-19

## Abstract

**Background:**

Neuromuscular electrical stimulation (NMES) has demonstrated its efficacy in improving strength, muscle development, optimising microcirculation, reducing frailty and mortality risk. A better understanding of its prescription and effects in patients with coronavirus syndrome post (COVID-19) could favour its use.

**Objectives:**

To find evidence that compares the effectiveness of NMES in the increasing muscle mass, muscle strength and functional independence of patients in post-intensive care unit (ICU) with COVID-19 syndrome.

**Method:**

A systematic search was carried out in electronic databases: PubMed, Science Direct, Scopus, Ovid and Cochrane from 22 May 2022 to 30 April 2023, without language restriction, including clinical controlled trials (CCTs) and prospective longitudinal studies (PLS). Prospero registration (CRD42022332036).

**Results:**

A total of 1718 scientific articles were found; four articles met the inclusion criteria. For NMES dosing, intervention time ranged from 9 days to 30 days, the stimulus frequency was between 20 Hz and 121 Hz and the pulse width was between 350 µs and 1400 µs. The application time ranged from 30 min to 60 min, the intensity was between 20 mA and 250 mA and the stimulated muscle groups were quadriceps, tibialis anterior, hamstrings and gluteus.

**Conclusion:**

The use of NMES after COVID-19 such as integral complementary strategy improves muscle mass, strength and functionality of the patients optimising recovery results.

**Clinical implications:**

The addition of NMES to standard physical therapy might have a positive impact on the recovery of individuals who have survived COVID-19.

## Introduction

At the end of 2019, a rapidly expanding health problem occurred worldwide caused by the severe acute respiratory syndrome coronavirus 2 (SARS-COV-2) virus. The World Health Organization (WHO) declared a health emergency at the international level (WHO 2022). Coronavirus disease 2019 (COVID-19) is known for its various signs and symptoms, which include muscle and body pain, headache, cough and nasal congestion, to developing serious respiratory pathologies that could even induce death (Ahmed et al. 2020).

A prolonged stay at the intensive care unit (ICU) was characteristic of patients who exhibited the severe form of the disease; in most of them, their stay was justified by the high mechanical ventilation (MV) requirements and the high predisposition to require higher doses of sedative drugs and muscle relaxants for their treatment adaptation and tolerance. This approach induced in patients with long periods of immobilisation triggered disuse muscle dysfunction and atrophy, muscle weakness and the critically ill patient’s physical deconditioning syndrome (Pérez Abreu, Gómez Tejeda & Dieguez Guach 2020). For those who survived this condition, multisystem sequelae of musculoskeletal, neuromuscular, cardiovascular, pulmonary, neurological and integumentary types are frequently reported, as well as reduced lung function and exercise capacity, post-traumatic stress disorder, depression, anxiety and reduced quality of life (Ahmed et al. 2020; The Novel Coronavirus Pneumonia Emergency Response Epidemiology Team 2020).

It has been demonstrated that physiotherapeutic intervention mitigates the appearance of sequelae in patients after COVID-19, thus reducing morbidity, mortality and hospital costs (Llamosas Falcón n.d.). The rehabilitation of these patients includes techniques such as aerobic capacity training and strength recovery; in addition, secretion drainage or ventilatory techniques are used, whose objective is to re-educate the respiratory pattern, improve ventilation, mobilise the thorax and favour secretion drainage (Sheehy 2020). Among the techniques described for the intervention of patients after COVID-19, neuromuscular electrical stimulation (NMES) appears as an alternative. This technique uses surface electrodes attached to the skin, transmitting an electrical current pulse of varying intensity to stimulate skeletal muscle (Manta et al. 2022). Some research reports the benefits of NMES to counteract the adverse effects of prolonged immobilisation (Stripari Schujmann & Annoni 2020), which can result in muscle atrophy and generalised weakness, common conditions in patients with prolonged stay in ICU, reducing hospitalisation time and hospital costs (Betancourt-Peña et al. 2022; Junqué Jiménez et al. 2014).

Despite the growing evidence of NMES as an alternative treatment to improve strength, muscle mass and functional independence in post-COVID patients (Cardona Pérez et al. 2014), its use in skeletal muscle presents wide variability in its application protocols including the intervention time, the location of the electrodes, the input and output times of the current, the type of impulse, the frequency (Hz), the pulse width (µs) and intensity (mA). In particular, NMES is commonly applied to muscles such as the quadriceps and hamstrings, where correct electrode placement is essential to optimise stimulation and thus counteract the muscle weakness and atrophy that often affect these patients. Given the widespread utilisation of this method among populations with neuromuscular deficiencies, research reports point to a global improvement in the functional condition of the individual (Sepúlveda-Loyola et al. 2022) to broaden knowledge regarding the prescription and effects of the application of NMES in patients with post-COVID-19 syndrome. This could guide the professionals in charge of its application to define the best form of implementation based on current scientific evidence. In this systematic review, we aimed to find evidence that compares the effectiveness of NMES vs. placebo in the increasing muscle mass, muscle strength and functional independence of patients with post-COVID-19 syndrome.

## Research methods and design

This systematic review followed the recommendations made by the *Cochrane Handbook for Systematic Reviews of Interventions* (eds. Higgins & Green 2011) and the PRISMA Statement 2020 (Page et al. 2021). The protocol for this study was registered in The International Prospective Register of Systematic Reviews (PROSPERO) [CRD42022332036].

### Eligibility criteria

We searched for studies where patients post-COVID-19 infection with epidemiological discharge and negative PCR were admitted to intensive care units because of complications or decompensation of chronic non-communicable disease with sequelae. We excluded studies with patients who had COVID-19 but were not rehabilitated with NMES and those who had a clinical history with underlying pathologies before being infected with COVID-19, such as skin lesions, cardiac pacemakers, infection or trauma to the extremities, neuromuscular diseases and use of neuromuscular blockers. We also excluded systematic reviews. The types of studies included were randomised clinical trials, case-control studies, cohort studies and prospective longitudinal studies, which allowed an analysis of the measurements before and after the application of NMES in order to establish the cause–effect relationship associated with the intervention.

### Information search sources

We conducted a systematic search in PubMed, ScienceDirect, Scopus, Ovid and the Cochrane Central Register of Controlled Trials (CENTRAL) between 22 May 2022 to 30 April 2023 without language restriction.

We used MeSH terms for the search and the different combinations with the Boolean connectors in the following equation:

(stimulation OR Electric Stimulation Therapy OR EEN OR NMES OR neuromuscular electrical stimulation OR electric stimulation OR Neuromuscular OR Rehabilitation OR therapeutics OR treatments OR therapy OR therapy OR treatment OR treatments OR treat* OR clinical trial OR clinical trials as topic OR clinical trials OR randomized controlled trial OR randomized controlled trials as topic OR randomized controlled trials OR therapeutics OR therapies OR therapy OR therapies OR therapeutic OR therapeutically OR therapeutics OR therapeutics OR therapeutic) AND (respiratory diseases OR acute respiratory distress OR pulmonary OR Lung OR Pulmonary Gas Exchange OR Pulmonary Diffusing Capacity OR COVID-19 OR post COVID-19 recovery OR coronavirus infection Coronavirus OR Respiratory Tract Infections OR SARS-Cov-2 OR Severe Acute Respiratory Syndrome Coronavirus 2 OR NCOV OR 2019 NCOV) AND (muscle contraction OR muscle spasm OR muscle reduction OR muscle twitching OR muscle shrinkage OR muscle wasting OR muscular retraction OR muscle contortion OR muscle pain) AND (status functional OR functional OR functional independence OR activities of daily living OR health status). Connectors were used in different combinations according to each database consulted to reach the largest number of published articles (Online Appendix 1, Table 1-A1).

### Selection of studies

One investigator collected data from the included articles, and two investigators analysed these data simultaneously using an instrument developed by the authors in an Excel matrix. Studies that met the inclusion criteria were identified, and duplicate records were eliminated. The first selection of studies was based on the title and abstract to specify whether the study or article addressed the topic in question and answered the research question. Subsequently, potential articles were read in full text to critically analyse the research characteristics. Once the information was extracted, the researchers confronted the agreement and disagreement regarding the content of the selected articles. In case of disagreement among the authors, discussions and consultations were conducted with the participation of a fourth author. This step also avoided the risk of selection bias in the included articles.

### Data extraction

The data extracted from the studies included information on COVID-19, NMES, muscle mass, muscle strength and functional independence. Primary outcome variables were muscle mass by ultrasonography (U) or cross-sectional area (CSA), muscle strength by surface electromyography (sMEG), ankle maximal voluntary isometric contractions (MVIC), manual grip strength (HGS), Medical Research Council (MRC) Scale, ICU physical function test (PFIT-s) and functionality of the lower extremities evaluation of the probability of falls through the Morse Fall Scale (MFS).

### Additional outcomes

We included the results regarding health-related quality of life assessed with a 36-item short form survey (SF-36), Morton Mobility Index (DEMMI), Intensive Care Unit Optimal Mobilization Score (SOMS), time spent sedentary lifestyle and time spent walking or running from the studies included.

### Assessment of quality

The PEDro scale was used to evaluate randomised clinical trials. This scale consists of 11 items where 1 is scored if it complies and 0 if it does not comply, and according to the sum of the score, it is determined if the methodological quality of the article is low, intermediate or high, taking into account aspects such as adequate control group, blinding and randomisation (Moseley et al. 2020). The Minors scale was used to evaluate observational studies (case-control studies, cohort studies and prospective longitudinal studies). This scale consists of 12 items (clearly defined objective, inclusion of patients consecutively, prospective data collection, results appropriate for the study objective according to the intention to treat, unbiased outcome assessment [blinding], follow-up period appropriate for study objective, loss to follow-up less than 5%, calculation of study sample size, 95% confidence interval, an adequate control group, groups managed at the same time both control and study, baseline equivalence of groups and adequate statistical analysis). Each item is assigned a score of 0 if it is not reported, 1 if it is reported but is inadequate and 2 if it is reported and is adequate. Once the points are added, it is established that the ideal score would be 16 for non-comparative studies and 24 for comparative studies (Slim et al. 2003).

### Data synthesis strategy

A qualitative summary of included study designs, population characteristics, number of participants in each study, COVID-19 diagnostic methods, measures of muscle mass, muscle strength and independence was provided.

#### Strategy for data analysis

For normally distributed continuous variables, we reported means, standard deviations and the number of participants in each group. From non-normally distributed continuous variables, we extracted medians and interquartile ranges. We also present confidence intervals and *p*-values. From dichotomic variables, we extracted proportions and percentages. Any discrepancy between the two investigators was resolved by discussion or consultation with other co-authors of the systematic review.

### Ethical considerations

Our study consists of secondary research; thus, ethical approval was not required for our systematic review.

## Results

**FIGURE 1:**
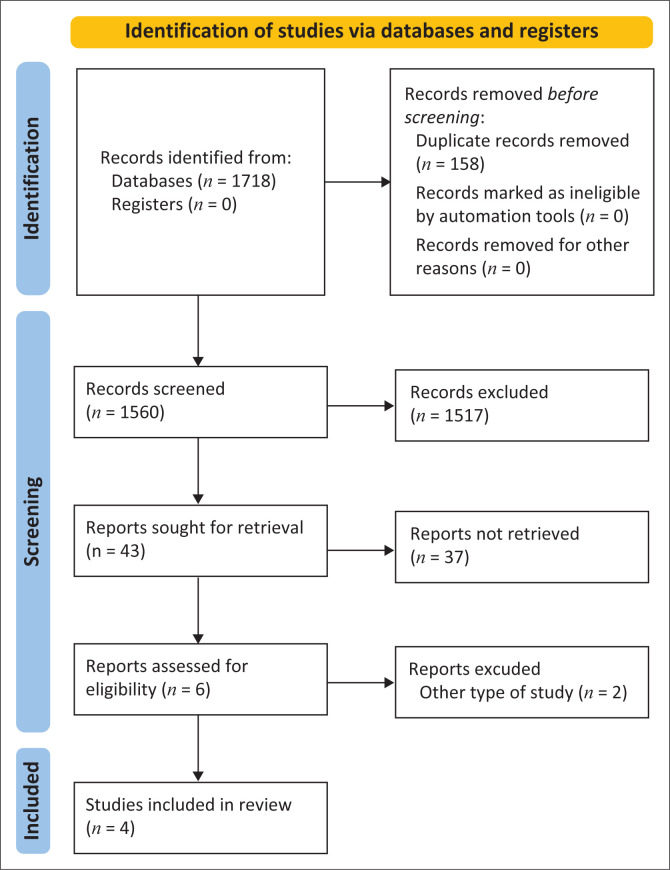
PRISMA flow diagram 2020.

The systematic review yielded 6488 studies from the five databases searched; before the selection phase, 3370 records were eliminated because of duplication. A total of 1560 records were evaluated by title and abstract, and 1517 records were excluded for not meeting the inclusion criteria. At the last stage of selection, two were excluded as they did not include outcome measures assessed in this research (Online Appendix 1, Table 2-A1). After reading full texts of eligible studies, four were selected for analysis in this systematic review (Figure 1).

### Characteristics of the studies included

Three of the included studies were controlled clinical trials (CCT), and one was a prospective longitudinal study (PLS); 50% were from the European continent (Table 1).

**TABLE 1:** Characteristics of the studies included.

No.	Reference	Type of study	Country	Year	Aim
1	Ozyemisci Taskiran et al. (2021)	CCT	Turkey	2021	To evaluate the effects of the early rehabilitation program in the intensive care unit on patients with acute respiratory distress syndrome secondary to COVID-19.
2	Righetti et al. (2022)	PLS	Brazil	2022	To assess the effects of the NMES intervention on muscle mass and functionality in patients with severe COVID-19 associated with sepsis and septic shock.
3	Mateo et al. (2021)	CCT	France	2021	To assess whether cycling with FES could be used safely in combination with physiotherapy soon after ICU discharge in critically ill COVID-19 patients and favour restoration of upright position on spontaneous resumption of gait more quickly than rehabilitation no FES.
4	Zulbaran-Rojas et al. (2022)	CCT	United States	2022	To examine the potential safety and efficacy of lower extremity NMES in preventing lower extremity muscle deconditioning in patients with COVID-19 in the ICU.

Note: Please see the full reference list of this article Carvajal-Tello, N., Segura-Ordóñez, A., García-Muñoz, H., Sánchez-Montoya, L.J., Cambindo-Larrahondo, L.M., Muñoz-Chaux, V. et al., 2025, ‘Systematic review effects of neuromuscular electrical stimulation in post-coronavirus disease’, *South African Journal of Physiotherapy* 81(1), a2132. https://doi.org/10.4102/sajp.v81i1.2132 for more information.

CCT, controlled clinical trial; PLS, prospective longitudinal study; NMES, neuromuscular electrical stimulation; FES, functional electrical stimulation; ICU, intensive care unit; COVID-19, coronavirus disease 2019.

### Participant characteristics

Table 2 presents the characteristics of the subjects. The total number of participants was 72, the participants were aged between 63 years and 73 years and 51% of the participants were men (*n* = 37). All four studies included men and women. The BMI of the patients was between 24.8 and 32.45 (Table 2).

**TABLE 2:** General characteristics of the patients.

No.	Reference	Total	EG and CG	Sex	Age (years)	BMI (kg/m^2^)	Comorbidities	Inclusion criteria	Exclusion criteria
1	Ozyemisci Taskiran et al. (2021)	*N* = 35 F (*n* = 11) M (*n* = 24)	Exposure group *N* = 18	F (*n* = 11, 61%) M (*n* = 7, 39%)	Median 73 (IQR 64–78)	Median 24.8 (IQR 19.5–28.1)	CVD (*n* = 5, 28%) DM (*n* = 6, 33%) HTN (*n* = 7, 39%) EPC (n = 8, 44%) Cancer (*n* = 6, 33%) Neurological disease (*n* = 4, 22%)	Patients admitted to the ICU with a diagnosis of ARDS secondary to COVID-19, age > 18 years. These critical cases were defined as respiratory failure requiring MV or shock or organ failure, requiring ICU care in accordance with the novel coronavirus pneumonia diagnosis and treatment protocol.	Failure to meet the following minimum medical stability criteria: T < 38.2 °C, HR > 60 bpm, < 120 bpm, RR < 30 breaths/minute, SBP > 90 mmHg, < 180 mmHg, SatO2 > 92%, absence vasopressor dose increase, no arrhythmia (except chronic atrial fissure), no > 50% progress on chest imaging within 24–48 h, Fio2) ≤ 0.6, PEEP ≤ 10 cmH_2_O.
			Control group *N* = 17	F (*n* = 4, 23%) M (*n* = 13, 77%)	Median 70 (IQR 62–76)	Median 29.4 (IQR 26.3–33.7)	CVD (*n* = 5, 29%) DM (*n* = 6, 35%) HTN CG= 10 (59%) EPC (*n* = 1, 6%) Cancer (*n* = 2, 12%), Neurological disease (*n* = 0, 0%)		
2	Righetti et al. (2022)	*N* = 7 F (*n* =2) M (*n* = 5)	Exposure group *N* = 7 (100%)		Median 68.1 ± s.d. 4.6	Median 30.2 ± s.d. 2.3	HTN (*n* = 4, 57%) DM (*n* = 1, 14%) Obesity (*n* = 4, 57%) EDL (*n* = 3, 42%) Anxiety (*n* = 0, 0%) Hypothyroidism (*n* = 2, 28%)	BMI ≤ 35 kg/m^2^, no skin lesions, cardiac pacemaker, infection or trauma in MI, neuromuscular diseases, use of neuromuscular blockers, polyneuropathy, and imminent risk of death in less than 48 h. (Simplified Acute Physiology III Score – SAPS III ≤ 80).	Infarction and the need for mechanical cardiopulmonary bypass devices or the need for an intra-aortic balloon during hospitalization in the ICU.
3	Mateo et al. (2021)		Exposure group *N* = 8	M (*n* = 7, 87%) F (*n* = 1, 12.5%)	Median 62.8 ± s.d. 9.1	Median 28.1 ± s.d. 4.8	CVD (*n* = 5, 63%) EPC (*n* = 1, 13%) Depression and anxiety XXX 5.1 (XXX3.2)	Hospices Rehabilitation Department Civils de Lyon during the first pandemic wave in France after hospitalization in the ICU for a critical form of COVID-19.	Cognitive deficit or neurological or psychiatric comorbidity.
		*N* = 14 F (*n* = 2) M (*n* = 12)	Control group *N* = 6	M (*n* = 5, 83%) F (*n* = 1, 16%)	Median 64.8 ± s.d. 7.0	Median 27.1 ± s.d. 4.4	CVD (*n* = 2, 33%) EPC (*n* = 0, 0%) Depression and anxiety XXX = 5.1 (XXX 3.2)		
4	Zulbaran-Rojas et al. (2022)	*N* = 16 F (*n* = 7) M (*n* = 9)	Exposure group *N* = 8	F (*n* = 5, 62.5%) M (*n* = 3, 37.5%)	Median 66.75 ± s.d. 9.81	Median 28.49 ± s.d. 7.17	CVD (*n* = 1, 12.5%) DM (*n* = 3, 37.5%) HTN (*n* = 5, 62.5%) EDL (*n* = 4, 50%) IRA (*n* = 1, 12.5) IRC (*n* = 1, 12.5%) Anemia (*n* = 8, 100%) Pneumonia (*n* = 7, 87.5%)	Patients admitted to the ICU because of COVID-19 infection within 3 days before NMES therapy, receiving assisted ventilation therapy and having indicated bed rest for at least 7 days.	Patients medically sedated or under vasopressor therapy, expected to be discharged from the ICU in the next 24 h; had below-knee amputations or lower extremity injuries; demand cardiac pacemaker, implanted defibrillator or other implanted electronic devices; and any condition that may interfere with the results or increase the risk of NMES use in the judgement of physicians.
			Control group *N* = 6	F (*n* = 2, 25%) M (*n*= 6, 75%)	Median 62.88 ± s.d. 9.51	Median 32.45 ± s.d. 8.04	CVD (*n* = 1, 12.5%) DM (*n* = 6, 75%) HTN (*n* = 6, 75%) EDL (*n* = 2, 25%) IRA (*n* = 1, 12.5) IRC (*n* = 2, 25%) Anemia (*n* = 6, 75%) Pneumonia (*n* = 8, 100%)		

Note: Please see the full reference list of this article Carvajal-Tello, N., Segura-Ordóñez, A., García-Muñoz, H., Sánchez-Montoya, L.J., Cambindo-Larrahondo, L.M., Muñoz-Chaux, V. et al., 2025, ‘Systematic review effects of neuromuscular electrical stimulation in post-coronavirus disease’, South African Journal of Physiotherapy 81(1), a2132. https://doi.org/10.4102/sajp.v81i1.2132 for more information.

F, female; M, male; EG, exposure group; CG, control group; N/E, not specified; CVD, cardiovascular diseases; DM, diabetes mellitus; HTN, hypertension; ARDS, syndrome of acute respiratory distress; MV, mechanical ventilation; ICU, intensive care unit; T, temperature; HR, heart rate; RR, respiratory rate; SBP, systolic blood pressure; SaO2, oxygen saturation; PEEP, positive end-expiratory pressure; LL, lower limbs; BMI, body mass index; CPD, chronic pulmonary disease; ARI, acute renal failure; CRF, chronic renal failure; EDL, pulmonary diffusion disease; PD, pulmonary disease; CD, heart disease; C, cancer; HLPDM, hyperlipidemia; NMES, neuromuscular electrical stimulation; ICU, intensive care unit; COVID-19, coronavirus disease 2019; EPC, chronic lung disease; IRA, acute respiratory failure; IRC, chronic kidney failure; SAPS, aimplified acute physiology score; FiO2, inspired fraction of oxygen; IQR, interquartile range; s.d., standard deviation.

### Dosage parameters for the application of the neuromuscular electrical stimulation

The intervention time ranged between 9 days and 30 days, while each session lasted 30–60 min. The muscle groups stimulated were mainly those of the lower limbs, including quadriceps, tibialis anterior, hamstrings and gluteal muscles. Regarding the dosage of the NMES parameters, the ON time was between 4 s and 6 s, and the OFF time was 12 s; biphasic waves were observed in the impulse type across all studies except for one study, which did not report them (Ozyemisci Taskiran et al. 2021). The frequency in Hz was between 20 and 121, the pulse width was between 350 µs and 1400 µs and the intensity was between 20 mA and 250 mA (Table 3).

**TABLE 3:** Dosage parameters for the application of the neuromuscular electrical stimulation.

No.	Reference	Intervention time/session duration	Electrode location	On/off time	Impulse type	Frequency (Hz)	Pulse width (µs)	Current (mA)	Intervention in CG
1	Ozyemisci Taskiran et al. (2021)	9 days / 52 min	Quadriceps and tibialis anterior muscles bilaterally.	6 s on / N/E sec off	Symmetrical biphasic square waves.	50	N/A	20–25	Standard ICU care = Medical monitoring and treatment.
2	Righetti et al. (2022)	7 days / 40 min	Vastus medialis lateralis muscles distally over the motor area and 5 cm below the inguinal region.	4 s on / 12 sec off	Balanced charge biphasic pulses and trapezoidal waves.	100	350	Awake patients = Maximum contraction tolerated per patient. Sedated patients = 50%.	N/A
3	Mateo et al. (2021)	30 days / 30 min	The quadriceps muscle, hamstrings, tibialis anterior and gluteus maximus or triceps surae.	N/E	N/E	N/E	N/E	Patients were instructed to maintain a plateau of at least 60% of the maximum stimulation intensity setting.	Cycling without NMES.
4	Zulbaran-Rojas et al. (2022)	14 days / 60 min	Gastrocnemius muscle and Achilles tendon.	N/E	Asymmetric damped sinusoidal biphasic pulsed wave.	20 and 121	400 and 1400	50–250	An identical but non-functional device (placebo) was provided for the same period.

Note: Please see the full reference list of this article Carvajal-Tello, N., Segura-Ordóñez, A., García-Muñoz, H., Sánchez-Montoya, L.J., Cambindo-Larrahondo, L.M., Muñoz-Chaux, V. et al., 2025, ‘Systematic review effects of neuromuscular electrical stimulation in post-coronavirus disease’, *South African Journal of Physiotherapy* 81(1), a2132. https://doi.org/10.4102/sajp.v81i1.2132 for more information.

N/E, not specified; N/A, not applicable; CG, control group; ICU, intensive care unit; PPS, pulses per second; NMES, neuromuscular electrical stimulation.

### Evaluation of the effect of neuromuscular electrical stimulation on the increase in muscle mass, muscle strength and functional independence

Regarding the increase in muscle mass, only one study reported evaluating it with measurement of the cross-sectional area and ultrasonography (Righetti et al. 2022); for the evaluation of muscle mass, one study evaluated it with manual grasping force (Mateo et al. 2021), and three studies measured it with the Medical Research Council Scale (MRC) (Mateo et al. 2021; Ozyemisci Taskiran et al. 2021; Righetti et al. 2022) finding for the experimental group between 47.7 (9.2) and 58 (50–60). The studies employed to evaluate functional independence, the SF-36 (Mateo et al. 2021), the time spent sedentary and walking or running (Ozyemisci Taskiran et al. 2021), the ICU physical function test (PFIT-s), the Morton Mobility Index (DEMMI), Optimal Mobilization Score of the Intensive Care Unit (SOMS) (Righetti et al. 2022) and lower extremity functionality evaluation of the probability of falls through the Morse Fall Scale (MFS) (Zulbaran-Rojas et al. 2022) (Table 4).

**TABLE 4:** Evaluation of the effect of the NMES on the increase in muscle mass, muscle strength, and functional independence.

No.	Reference	Measurements	EG and CG	Basal	Later	Observations
1	Ozyemisci Taskiran et al. (2021)	PMF (kg)	Exposure group	N/A	Median 30 (IQR: 13–32)	The study reports the evaluations before applying NMES; however, it does not report the base values. He mentions that hemodynamic instability was not observed in the application and that the duration of MV, mortality, and stay in the ICU and hospital were more significant in the EG, although the difference was not significant.
			Control group		Median 22 (IQR: 11–27)	
		MRC (score)	Exposure group	N/E	Median 58 (IQR: 50–60)	
			Control group		Median 57 (IQR: 51–60)	
		SF-36	Exposure group	N/E	Median 90 (IQR: 38–100)	
			Control group		Median 75 (IQR: 45–80)	
2	Righetti et al. (2022)	CSA (cm2)	-	Quadriceps Day 1= Median 1.44 (IQR: 1.20–1.68) Rectus Femoris Day 1= Median 0.65 (IQR: 0.34–0.87)	Quadriceps Day 8= Median 1.43 (IQR: 1.20–1.65) Rectus Femoris Day 8 = Median 0.52 (IQR: 0.18–0.85)	CSA of the rectus femoris decreased significantly (–16.9% [95% CI, –29.8 to –3.9]; *p* < 0.05) from days one to eight but showed maintenance of the thickness of the rectus femoris. Anterior compartment of the quadriceps muscle (-3.20% [95% CI, –10.6 to 4.2]; *p* = 0.3) from days 1 to 8. These patients showed a 2.1% reduction [95% CI: –3.7 to –0.5] per day in the cross-sectional area of the rectus femoris and 0.3% [95% CI: –1.3 to 0.5] per day in the thickness of the anterior compartment of the quadriceps muscle for eight days. In addition, patients showed maintenance of echogenicity (1.3% [95% CI, –17.1 to 19.7%]; *p* = 0.8) from days one to eight, with an increase of 0.16 % per day.
		U (pixels)	-	Rectus Femoris Day 1 = Median 85.7 (IQR: 52.3–117.5)	Rectus Femoris Day 8 = Median 75.6 (IQR: 55–97)	
		MRC (score)	-	Day 1 = Median 18.8 (IQR: 5.3–43.6)	Day 8 = Median 52.4 (IQR: 49.2–55.8)	Four (80%) tested patients showed increased MRC scores; one (20%) maintained MRC score values from days five to eight.
		PFIT-s (score)	-	Day 1 = Median 1.63 (IQR: 0.7–3.4)	Day 8 = Median 6.6 (IQR: 4.9–8)	They increased significantly from days one to five and improved through day eight compared to day five (*p* < 0.05)
		DEMMI (score)	-	Day 1 = Median 7.4 (IQR: 4.9–19)	Day 8 = Median 45 (IQR: 21.1–68.3)	DEMMI and SOMS scores were significantly increased on day eight compared to days one and five (*p* < 0.05).
		SOMS (score)	-	Day 1= Median 0.74 (IQR: 0.3–1.7)	Day 8= Median 4 (IQR: 3.8–4.2)	
3	Mateo et al. (2021)	MRC (score)	Exposure group	Median 47.7 ± s.d. 9.2	N/E	The data was taken at the beginning of the intervention; it does not report subsequent data from the MRC scale.
			Control group	Median 50.2 ± s.d. 4.8	-	
		Time spent sedentary	Exposure group (cycling and FES)	Median 778.6 ± s.d. 30.2	Median 148.3 ± s.d. 58.5	FES cycling was associated with a greater beneficial decrease in daytime time spent sedentary (e.g., lying down, reclining, or sitting): 200.8 min (95% CI: 363.5, 38.2; *p* < 0.02) size of the effect of 0.54 (moderate effect). (effect size = 0.52).
			Control group (cycling)	Median 778.6 ± s.d. 30.2	Median 52.5 ± s.d. 70.9	
		Time spent walking or running	Exposure group (cycling and FES)	Median 6.8 ± s.d. 3.8	Median 43.5 ± s.d. 6.8	The FES cycling group showed a greater increase in time spent walking or running: 22.2 min (95% CI: 2.5, 41.9 min; *p* < 0.03)
			Control group (cycling)	Median 6.8 ± s.d. 3.8	Median 21.3 ± s.d. 8.3	
4	Zulbaran-Rojas et al. (2022)	MVIC (kg)	Exposure group	Mean 2.7 ± s.d. 1.7	Mean 3.0 ± s.d. 1.6	At three days, the EG showed a non-significant improvement compared to the CG with mean effect sizes for Ankles (*p* = 0.06, d = 0.77)
			Control group	Mean 2.5 ± s.d.1.2	Mean 2.1 ± s.d. 0.7	
		sMEG	Exposure group	Mean 331 ± s.d. 10	Mean 338 ± s.d. 36	The CG showed a non-significant impairment for sMEG compared to baseline (–3.9%, *p* = 0.08). At nine days, the EG showed a significant improvement compared to the CG, with a large effect size for sMEG (*p* = 0.04; d = 0.97). EG sMEG showed a significant improvement (+6.3%, *p* = 0.029) compared to the baseline.
			Control group	Mean 327 ± s.d. 12	Mean 314 ± s.d. 27	
		MFS	Exposure group	Mean 43.7 ± s.d.17	Mean 39.3 ± s.d. 11	Compared with the baseline, the EG MFS score showed a significant improvement (–12.7%, *p* = 0.05), in contrast to the CG, which showed a significant worsening score (48.1 %, *p* = 0.04).
			Control group	Mean 31.2 ± s.d. 7.4	Mean 46.2 ± s.d. 11.8	

Note: Please see the full reference list of this article Carvajal-Tello, N., Segura-Ordóñez, A., García-Muñoz, H., Sánchez-Montoya, L.J., Cambindo-Larrahondo, L.M., Muñoz-Chaux, V. et al., 2025, ‘Systematic review effects of neuromuscular electrical stimulation in post-coronavirus disease’, *South African Journal of Physiotherapy* 81(1), a2132. https://doi.org/10.4102/sajp.v81i1.2132 for more information.

CSA, cross-sectional area; U, ultrasonography; sMEG, surface electromyography; MVIC, ankle maximal voluntary isometric contractions; HGS, manual grip strength; MRC, Medical Research Council Scale; PFIT-s, physical function test of the ICU; DEMMI, Morton Mobility Index; SOMS, Optimal Mobilization Score of the Intensive Care Unit; MFS, functionality of the lower extremities evaluation of the probability of falls through the Morse Fall Scale; NMES, neuromuscular electrical stimulation; ICU, intensive care unit; N/E, not specified; N/A, not applicable; EG, exposure group; CG, control group; PMF, hand grip strength; FES, functional electrical stimulation; MV, mechanical ventilation; IQR, interquartile range; s.d., standard deviation.

### Methodological quality

The methodological quality was evaluated with the PEDro score for three studies whose study type corresponded to CCTs, and a PLS was evaluated with the Minors score. The mean PEDro score for the CCTs, as described in Table 5, was intermediate for two studies and high for one study; the most frequent omissions in the study design or its reporting were the following: the randomisation process did not conceal and no blinding of subjects and assessors.

**TABLE 5:** Methodological quality of the controlled clinical trial studies (PEDro score).

No.	Reference	PEDro scale criteria											Total	Quality
		1†	2	3	4	5	6	7	8	9	10	11		
1	Ozyemisci Taskiran et al. (2021)	-	1	0	1	0	0	0	1	1	1	1	6	Intermediate
3	Mateo et al. (2021)	-	1	0	1	0	0	0	1	1	1	1	6	Intermediate
4	Zulbaran et al. (2022)	-	1	1	1	1	1	1	1	1	1	1	10	High

Note: Please see the full reference list of this article Carvajal-Tello, N., Segura-Ordóñez, A., García-Muñoz, H., Sánchez-Montoya, L.J., Cambindo-Larrahondo, L.M., Muñoz-Chaux, V. et al., 2025, ‘Systematic review effects of neuromuscular electrical stimulation in post-coronavirus disease’, *South African Journal of Physiotherapy* 81(1), a2132. https://doi.org/10.4102/sajp.v81i1.2132 for more information. PEDro scale criteria: (1) Selection criteria were specified, (2) subjects were randomly assigned to groups (in a crossover study, subjects were randomised as they received treatments), (3) allocation was concealed, (4) groups were similar at baseline with respect to major prognostic indicators, (5) all subjects were blinded, (6) all therapists who administered the therapy were blinded, (7) all raters who measured at least one key outcome were blinded, (8) measurements of at least one of the key outcomes were obtained from more than 85% of subjects initially assigned to groups, (9) results were presented for all subjects who received treatment or were assigned to the control group, or where this could not be, data for at least one key outcome were analysed by ‘intention to treat’, (10) the results of statistical comparisons between groups were reported for at least one key outcome and (11) the study provides point and variability measures for at least one key outcome. Score: 1 = item met, 0 = item not met. Quality criteria: ≥ 7 high quality, 5–6 intermediate quality and ≤ 4 low quality.

† , this item was not used to calculate the PEDro score.

The mean Minors score for a single PLS was 15 points (Table 6), considered high if one considers that the ideal score would be 16 for non-comparative studies. The methodological limitation of the study was associated with the non-biased evaluation of the results (blinding); for the methodological quality assessments with PEDro and Minors, an agreement between three raters was achieved without requiring the support of a fourth person.

**TABLE 6:** Methodological quality of the comparative studies (cohorts and cases and controls) Minors scale.

No.	Reference	Minors scale criteria												Total
		1	2	3	4	5	6	7	8	9	10	11	12	
2	Righetti et al. (2022)	2	2	2	2	0	2	2	1	0	0	0	2	15

Note: Please see the full reference list of this article Carvajal-Tello, N., Segura-Ordóñez, A., García-Muñoz, H., Sánchez-Montoya, L.J., Cambindo-Larrahondo, L.M., Muñoz-Chaux, V. et al., 2025, ‘Systematic review effects of neuromuscular electrical stimulation in post-coronavirus disease’, *South African Journal of Physiotherapy* 81(1), a2132. https://doi.org/10.4102/sajp.v81i1.2132 for more information. Minors scale criteria: (1) Clearly defined objective, (2) inclusion of patients consecutively, (3) prospective data collection, (4) results appropriate for the study objective according to the intention to treat, (5) unbiased outcome assessment (blinding), (6) follow-up period appropriate for study objective, (7) loss to follow-up less than 5%, (8) calculation of study sample size, 95% confidence interval, (9) an adequate control group, (10) groups managed at the same time both control and study, (11) baseline equivalence of groups, (12) adequate statistical analysis. Score: 0 = not reported, 1 = reported but inadequate, 2 = reported and adequate. The ideal score would be 16 for non-comparative studies and 24 for comparative studies.

## Discussion

We conducted an exhaustive search and identified four articles that met the inclusion criteria and employed the application of the NMES as a treatment measure for the recovery of patients in the post-COVID-19 stage and where an improvement was reported in muscle strength, functionality, the resumption of walking, the prevention of physical deconditioning and the improvement of strength in the respiratory muscles. This is consistent with the application of this type of intervention in critical patients in the ICU with various pathologies, as reported by Gruther et al. (2010), Dos Santos et al. (2020) and Gerovasili et al. (2012), showing an efficient recovery with the application of NMES in their studies, presenting positive results. NMES is a valuable complement to reverse atrophy and maintain muscle mass in patients who are in critical condition.

Regarding the application of the treatment in the four studies, it is possible to demonstrate dosage parameters for the NMES frequency between 20 Hz and 121 Hz (Zulbaran-Rojas et al. 2022), with pulse width between 350 µs (Righetti et al. 2022) and 1400 µs (Zulbaran-Rojas et al. 2022). The intervention time ranged from 9 days to 30 days of treatment; the location of the electrodes was mainly in the muscles of the lower limbs, such as quadriceps, tibialis anterior-posterior, vastus medialis – lateralis and hamstrings. Other studies have found similar findings on dosing parameters. For instance, in the article by Bao et al. (2022) on the prevention of muscle atrophy in the ICU with NMES, a pulsed current and a biphasic, asymmetric and balanced rectangular waveform was applied to the gastrocnemius and tibialis anterior muscles, with 30 Hz frequency, 300 µs wavelength and the value of time to stimulate the motor nerve, adjusting the intensity to the patient’s tolerance, 20 min, two times a day, for 7 days. In conclusion, they suggested that in addition to physical training, early application of NMES can prevent muscle atrophy. Meanwhile, the study by Kho et al. (2012) proposed an NMES protocol for ICU-acquired muscle weakness, based on the scientific literature for the quadriceps and gastrocnemius muscles with a pulsed, asymmetric current, balanced rectangular waveform, frequency of 50 Hz.

On the other hand, Cárdenas Favela et al. (2022) carried out a study in patients hospitalised in the ICU, where NMES therapy was applied to treat MV-induced diaphragmatic atrophy. The dosage parameters were a frequency of 30 Hz for mild and intermittent muscle contraction and a pulse width of 250 µs, allowing greater tolerance and assistance in muscle contraction. The proposed intervention time was 3 days, and the location of the electrodes was in the diaphragm, which focused on the recovery of respiratory muscles from MV-induced diaphragmatic atrophy.

Regarding ON and OFF times, two of the four studies in our review mentioned similar time ranges for ON time. The study by Ozyemisci Taskiran et al. (2021) showed an ON time interval of 6 s, but the OFF time was not specified. Compared to the article by Righetti et al. (2022), the time interval was 4 s ON, and the OFF time was 12 s. Comparing the dosage of NMES with the study by Mondragon, Ferrer and Quintero (2015), whose objective was to determine the joint effectiveness of NMES and early conventional therapy in patients with MV in the ICU, they proposed 8 s ON and 3 s OFF and the location of the electrodes in the muscles of the lower limb quadriceps, tibialis anterior and gastrocnemius. The study by Ulutaş, S N Öztekin and Ardıç (2021) also differed in the ON and OFF times implemented, where 10 s ON and 50 s OFF are described. This study consisted of a case report on the role of rehabilitation in a COVID-19 survivor with weakness acquired in the ICU, where NMES was performed as part of the intervention for 5 days a week with a duration of 20 min. This shows that the NMES dosage can be different according to each proposed intervention protocol determined by the researchers according to their clinical criteria.

Regarding the intensity of NMES in our study, it was set to patient tolerance for two studies (Ozyemisci Taskiran et al. 2021; Righetti et al. 2022), while Ozyemisci Taskiran et al. (2021) managed between 20 mA – 25 mA, and Zulbaran-Rojas et al. (2022) used between 50 mA – 250 mA, concluding in the studies the need for the use of sufficient intensity to obtain at least a visible contraction during application. On the contrary, Gerovasili et al.’s (2012) investigation had variable intensities regarding the prescription, managing ranges between 19 mA and 55 mA for the quadriceps and between 23 mA and 60 mA for the peroneal muscles. Another study by De Campos Biazon et al. (2021) showed that the average intensities should be kept between 29 mA and 33 mA.

It should be noted that, during the selection process of the articles included in this review, two studies were found with proposals for NMES protocols for COVID-19 patients, which were not included in the analysis as, being protocols, they did not show intervention results. The first study was that of Minetto et al. (2021), an intervention dosing parameter for a 15-day timeframe is suggested, with the duration of the session 30 min, location of the quadriceps and gastrocnemius muscle electrodes, ON and OFF times not specified, type of impulse symmetrical biphasic rectangular waves, frequency 30 Hz, pulse width 400 µs and intensity 135 mA. The second study was that of Kumar et al. (2022), which posed the following parameters: intervention time 9 days, 30-min session duration, application on the quadriceps muscle, times ON 15 min and OFF 15 min, type of impulse biphasic pulses, frequency 50 Hz, pulse width 400 µs and intensity according to sensory tolerance. The similarity in parameters within the NMES dosing protocols proposed by these two studies is apparent, and those found in the present review confirm this.

In our study, different evaluation methods were used to measure the results of the NMES, which did not allow for a correlation of the effect on the increase in mass, muscle strength and functional independence of the NMES. The only study that evaluated cross-sectional area by ultrasonography was the study by Righetti et al. (2022), while the study by Zulbaran-Rojas et al. (2022) used surface electromyography (sMEG) and maximum voluntary isometric ankle contractions. For its part, manual grasping strength was evaluated by the study by Ozyemisci Taskiran et al. (2021). The MRC scale was the most used, and three of the four included studies reported its measurement (Mateo et al. 2021; Ozyemisci Taskiran et al. 2021; Righetti et al. 2022). Other studies carried out on critically ill patients by Medrinal et al. (2021) and Burgess et al. (2021) also agree with using the MRC scale to evaluate muscle strength after applying NMES. In the study by Chen et al. (2019), the grip strength was evaluated in patients with COPD in the ICU, where the control group on the seventh day had a significant increase, whereas the intervention group did not see it reflected in the same way. In the patients included in the studies above, the incidence of muscle weakness caused by the prolongation of the hospital stay in the ICU and the requirement of mechanical ventilation is observed, where the objective of the intervention with NMES was in favour of increasing the intensity of early rehabilitation to prevent ICU-acquired atrophy.

For the evaluation of functionality, the studies included in this review also showed significantly different evaluations from each other, which also did not allow for establishing correlations. The study by Ozyemisci Taskiran et al. (2021) evaluated the SF-36 General Health Scale. In contrast, the study by Mateo et al. (2021) evaluated the time spent walking and running; the study by Righetti et al. (2022) counted with the physical function scale of the ICU (PFIT-s), the Morton Mobility Index (DEMMI) and the Optimal Mobilization Score of the Intensive Care Unit (SOMS); and the study by Zulbaran-Rojas et al. (2022) evaluated the functionality scale of the lower extremities. Koçan Kurtoğlu et al.’s (2015) study findings, which evaluated the effect of NMES in the prevention of ICU-acquired weakness in patients with COPD, coincided with our study in the form of evaluation with the SF-36 scale, showing an improvement significantly in physical function, vitality and social function.

A limitation of our study was the number of studies analysed as, after the methodological rigour applied in the review, only four studies were included with results that allowed us to analyse the effectiveness of the NMES in post-COVID-19 patients. However, the recent outbreak of COVID-19 and the narrow scientific production time frame must be considered. Even the effects of NMES and its dosage for the improvement of post-COVID patients are the subject of research worldwide. Additionally, it was not possible to compare the functional evaluations of the studies because no similarity was found in the selection of the measurement instruments. On the contrary, the dosage of the NMES prescription parameters was different in each protocol, which does not allow for making recommendations for specific parameters during the intervention, most likely because of the different comorbidities and conditions of the patients in critical care. However, all the analysed investigations showed positive changes in the recovery after the technique used. Although most of the selected studies had adequate methodological quality, showing that NMES can maintain or increase muscle strength, maintain muscle mass and volume and increase independence for activities of daily living, more research is still needed to include the benefits of optimal early rehabilitation, focused on post-COVID19 patients and surviving ICU patients.

Despite its limitations, this systematic review provides objective information on the application of the NMES in post-COVID-19 patients. Future research could be focused on evaluating the effects of this intervention in reducing patient mortality, hospital stay or functionality. Although some articles have described these variables, they are still the subject of research.

## Conclusion

The use of EEM as part of a comprehensive approach in post-COVID-19 rehabilitation not only improves muscle mass and strength but also enhances the patient’s overall functionality. Therefore, its implementation can be considered a valuable strategy to optimise recovery outcomes in these patients.
